# eHealth Interventions Targeting Poor Diet, Alcohol Use, Tobacco Smoking, and Vaping Among Disadvantaged Youth: Protocol for a Systematic Review

**DOI:** 10.2196/35408

**Published:** 2022-05-13

**Authors:** Lyra Egan, Lauren Anne Gardner, Nicola Newton, Katrina Champion

**Affiliations:** 1 The Matilda Centre for Research in Mental Health and Substance Use University of Sydney Sydney Australia

**Keywords:** eHealth, adolescent, health promotion, diet, alcohol, smoking, vaping, socioeconomic status, remoteness, rural, disadvantage

## Abstract

**Background:**

Chronic disease burden is higher among disadvantaged populations. Preventing lifestyle risk behaviors such as poor diet, alcohol use, tobacco smoking, and vaping in adolescence is critical for reducing the risk of chronic disease and related harms in adolescence and adulthood. Although eHealth interventions are a promising prevention approach among the general population, it is unclear whether they adequately serve adolescents from disadvantaged backgrounds such as those living in geographically remote or lower socioeconomic areas.

**Objective:**

This is the first systematic review to identify, evaluate, and synthesize evidence for the effectiveness of eHealth interventions targeting adolescents living in geographically remote or lower socioeconomic areas in preventing poor diet, alcohol use, tobacco smoking, and vaping.

**Methods:**

A systematic search will be conducted in 7 electronic databases: the Cochrane Database of Systematic Reviews, Cochrane Central Register of Controlled Trials, PROSPERO, MEDLINE (Ovid), Embase (Ovid), Scopus, and PsycInfo (Ovid). The search will be limited to eHealth-based experimental studies (ie, randomized controlled trials and quasi-experimental studies) targeting diet, alcohol use, tobacco smoking, and vaping among adolescents (aged 10-19 years). Eligible studies will be those reporting on at least one marker of socioeconomic status (eg, social class, household income, parental occupation status, parental education, and family affluence) or geographical remoteness (eg, living in rural, regional, and remote areas, or living outside major metropolitan centers). One reviewer will screen all studies for eligibility, of which 25% will be double-screened. Data will be extracted and summarized in a narrative synthesis. Risk of bias will be assessed using the Cochrane Revised Risk of Bias Tool.

**Results:**

As of December 2021, the title and abstract screening of 3216 articles was completed, and the full-text review was underway. The systematic review is expected to be completed in 2022.

**Conclusions:**

This systematic review will provide an in-depth understanding of effective eHealth interventions targeting poor diet, alcohol use, tobacco smoking, and vaping among adolescents living in geographically remote or lower socioeconomic areas and the factors that contribute to their effectiveness. This in turn will provide critical knowledge to improve future interventions delivered to these populations.

**Trial Registration:**

PROSPERO CRD42021294119; https://www.crd.york.ac.uk/prospero/display_record.php?RecordID=294119

**International Registered Report Identifier (IRRID):**

PRR1-10.2196/35408

## Introduction

Chronic disease burden is considerably higher among disadvantaged populations such as those living in lower socioeconomic or geographically remote areas [1-6]; therefore, disadvantaged adolescent populations may be vulnerable to experiencing greater chronic disease burden in adulthood than their counterparts. Socioeconomic status is an indicator of an individual’s or group’s social and economic position within society and is generally associated with access to resources and health outcomes [7]. In Australia, geographically remote refers to areas outside of major cities, classified in order of remoteness and decreasing level of accessibility to services as *inner regional*, *outer regional*, *remote*, or *very remote*. According to estimates from 2017 to 2018, 1 in 5 Australians have multiple chronic conditions, and almost half (45.1%) of those with multimorbidity aged ≥18 years live in the lowest 2 socioeconomic areas, compared to 15.2% in the highest socioeconomic area [8]. Moreover, the prevalence of multimorbidity is greater among populations living in *inner and outer regional* areas than in *major cities* (21% and 18%, respectively). This pattern is not unique to Australia, with similar sociodemographic differences in multimorbidity reported in several other high-income countries [9-14]. Living with a chronic condition impacts an individual’s quality of life and is accompanied with social and economic costs; this effect is amplified with multimorbidity [15,16]. Health inequity among disadvantaged populations may partially be explained by a degree of disadvantage pertaining to access to health and support services, as well as education and employment opportunities [17,18]. However, targeting lifestyle differences at a community level to reduce the vulnerabilities of disadvantaged populations [19] may provide positive benefits to overall health and narrow the inequalities in health.

Importantly, many chronic diseases share common lifestyle risk factors that are modifiable, such as poor diet, alcohol use, tobacco smoking, and vaping (electronic cigarette or “e-cigarette” use) [1,20-25]. Thus, reducing or avoiding the engagement in such behaviors can reduce total burden of disease figures. For example, in Australia, 38% of the total burden of disease in 2018 could have been prevented by reducing or avoiding the engagement in modifiable lifestyle behaviors [26]. However, engagement in these behaviors is not uniform across populations, with disparities existing between populations of different socioeconomic positions and between major cities and geographically remote areas. According to the *Australia’s Children Report* [27], children and adolescents living in the lowest socioeconomic area compared to those living in the highest socioeconomic area had the following differences: children aged 5-14 years were less likely to meet recommended fruit guidelines (63% compared to 74%) and more likely to consume sugar-sweetened beverages (SSBs) at least once a week (53% compared to 33%); and adolescents aged 12-14 years were more likely to consume alcohol at risky levels (2.2% compared to 0.1%) and be current smokers (2.9% compared to 1.4%). Although not a nationally representative sample, similar sociodemographic differences in diet and alcohol and tobacco use were observed in a recent, large study of 6640 children aged 11-14 years across Australia [28]. Specifically, students of lower socioeconomic status were more likely to use alcohol and tobacco and have poorer diets than students of middle to upper socioeconomic status, and students from regional areas were more likely to use alcohol than students from major cities. Similar sociodemographic differences in diet and alcohol and tobacco use have been reported among adolescents overseas [29-32]. Although vaping has historically been relatively uncommon in Australia, its prevalence has increased over the past decade [33,34]. According to the 2019 National Drug Strategy Household Survey, there has been a significant increase in e-cigarette use among people aged ≥14 years (11.3% in 2019 compared to 8.8% in 2016), with 14.5% of adolescents aged 14-19 years reporting lifetime use of e-cigarettes [33]. Almost half (49.3%) of adolescents aged 14-19 years had never smoked a tobacco cigarette before e-cigarette use. Its use is becoming more common among youth in other countries [35-37], with recent US data from the National Youth Tobacco Survey of 27 million high and middle school students finding that 27.5% (4.1 million) of high school students and 10.5% (1.2 million) of middle school students reported recent use [38]. Moreover, data from the UK Household Longitudinal Study found that e-cigarette use was greater among socioeconomically disadvantaged youth, particularly among never-smokers [39], and the 2018-2019 Kansas Communities That Care Student Survey—a large, cross-sectional, school-based survey of middle and high school students in Kansas (N=132,803)—found that adolescents from rural areas were more likely to report current e-cigarette use than those living in urban areas [40]. A recent meta-analysis of 23 studies found that among people aged <20 years, e-cigarette use triples the risk of initiating tobacco smoking [41]. Although not all individuals who use e-cigarettes will progress to tobacco smoking, they are at risk of experiencing e-cigarette or vaping-associated lung injury [42,43]. Therefore, it is important to consider vaping during adolescence as a chronic disease risk factor.

To reduce the risk of chronic disease in adulthood and address the disproportionately higher rates of chronic disease burden experienced by disadvantaged populations, targeting these behaviors prior to their onset and entrenchment is crucial [44,45]. For a young person, adolescence tends to be a period marked by greater autonomy over their life, as well as increased risk-taking behavior [46-48]. It is a time period in which experimenting with and using alcohol, tobacco smoking, and vaping generally increase [47,49-51]. Moreover, eating habits typically include greater purchasing of fast food away from home [52], along with an increased intake of nutrient-poor food [53], such as discretionary food items (eg, hot chips) [54] and SSBs [55]. These behaviors typically co-occur [56-59] and have been referred to as “consumption behaviors” [60], reflecting that individuals actively consume food, alcohol, or tobacco. In the short-term, these behaviors are linked to detrimental impacts such as poorer quality of life [61], behavioral and mental health issues [62,63], and obesity [64,65]. Several of these behaviors may track into adulthood [66,67], heightening the individual’s risk of chronic disease and associated burden over their lifetime, especially when they co-occur [20,68-70]. Altering this trajectory through the engagement of health promoting behaviors during adolescence shows promise in improving both adolescent and adult health outcomes [71].

Using eHealth interventions (eg, computer-, web-, mobile-, or telephone-based) is an approach with evidence to support its efficacy in targeting multiple risk behaviors in adolescents [72]. Given that eHealth interventions are delivered via the internet, they confer the advantages of increased implementation fidelity, cost-effectiveness, and accessibility, as well as improved student engagement [73,74]. Previous systematic reviews of eHealth interventions targeting at least one of the 4 aforementioned behaviors among adolescents have found them effective in the following areas: improving dietary behavior (eg, eating less unhealthy foods, lowering consumption of total fat and saturated fat, and significantly increasing daily fruit and vegetable intake) [75]; reducing alcohol use [76,77]; and reducing the number of cigarettes and smoking frequency [78]. These reviews, however, focused on adolescents in the general population and did not include vaping as one of the targeted behaviors. It is unclear whether eHealth interventions adequately serve adolescents living in geographically remote or lower socioeconomic areas and are effective in preventing vaping among these populations.

The purpose of this review is to identify, evaluate, and synthesize evidence for the effectiveness of eHealth interventions targeting adolescents (aged 10-19 years) from disadvantaged backgrounds in preventing poor diet, alcohol use, tobacco smoking, and vaping. Considering the personal, social, and economic burden attributed to poor diet, alcohol use, tobacco smoking, and vaping, particularly among disadvantaged populations, this systematic review will contribute valuable insights to the knowledge base and ideally guide the future development of effective eHealth interventions. To our knowledge, this is the first systematic review to focus specifically on eHealth interventions targeting poor diet, alcohol use, tobacco smoking, and vaping among adolescents with lower socioeconomic backgrounds or living in geographically remote areas.

## Methods

### Guidelines and Registration

This protocol conforms to the PRISMA-P (Preferred Reporting Items for Systematic Reviews and Meta-Analysis Protocols) guidelines [79] (see Multimedia Appendix 1) and was prospectively registered with PROSPERO (CRD42021294119).

### Eligibility Criteria

The population, interventions, comparators, and outcomes approach was used to address the research question and eligibility criteria for this review [79].

#### Population

Eligible studies will be human research studies that target adolescents aged 10-19 years, which aligns with the World Health Organization’s definition of “adolescent” [80]. Based on the demographic data presented by authors, studies will be eligible if the sample comprises any of the following: participants with lower socioeconomic status; participants living in rural, regional, or remote areas; and specific sub-group analysis among disadvantaged adolescents. Due to varying methods for measuring and defining socioeconomic status (eg, using the Family Affluence Scale III or Socio-Economic Indexes for Areas scores) and geographical remoteness (eg, based on relative access to services or termed as “countryside,” “village,” or “remote”), studies will not be limited to using the same measures or definitions; instead, socioeconomic status and geographical remoteness will be based on how they are conceptualized within the studies.

#### Intervention

Included studies will be those evaluating an eHealth intervention (eg, computer-, web-, mobile-, or telephone-based) targeting at least one consumption behavior—poor diet, alcohol, tobacco smoking, or vaping—among adolescents with lower socioeconomic status or living in geographically remote areas. Interventions addressing other risk behaviors in addition to poor diet, alcohol use, tobacco smoking, and vaping, such as poor sleep, sedentary screen time, and physical inactivity, will be eligible for inclusion as they may help to identify whether targeting a combination of certain behaviors influences outcomes.

#### Comparators

Eligible studies will compare the experimental group to a control group (eg, no intervention, education as usual, or an alternative intervention) or compare the changes in outcomes over time.

#### Outcomes

Primary outcomes of interest will include the reduced uptake or use of alcohol, tobacco, and vaping and improved or maintained dietary behaviors. Dietary behaviors will include any dietary outcomes, such as consumption of fruit and vegetables, SSBs, and nutrient-poor foods (junk food). Secondary outcomes of interest will include knowledge about diet, alcohol and tobacco use, alcohol-related harms, future intention to adopt health-related behaviors, motivators and barriers to adopting health-related behaviors, and other health behaviors such as sleep, sedentary screen time, and physical activity.

#### Studies

Included studies will be randomized controlled trials (including cluster randomized controlled trials) and quasi-experimental studies. They must be published in English and report original empirical findings. No date range restrictions apply for the included studies.

### Search Strategy

A database search strategy was developed in consultation with a research librarian. Searches will be conducted in the Cochrane Database of Systematic Reviews, Cochrane Central Register of Controlled Trials, PROSPERO, MEDLINE (Ovid), Embase (Ovid), Scopus, and PsycInfo (Ovid). The searching strategy to be used for all electronic databases is provided in Multimedia Appendices 2-8. Study references will be imported into EndNote software (Clarivate) and duplicates will be removed prior to being uploaded to Covidence software (Covidence) for screening. Grey literature websites and resources (eg, World Health Organization), conference abstracts, the reference lists of eligible studies, book chapters, and unpublished works (eg, dissertations and theses) will also be searched to identify any additional studies.

### Data Extraction and Screening

To balance rigor with the timeliness of the review, all study titles and abstracts will be screened by one reviewer (LE) against eligibility criteria, with a subset (25%) of studies double-screened by a second reviewer (NN, LAG, or KC). This method has been used in several systematic reviews [72,81,82]. Data will be extracted by one reviewer (LE) and reviewed by a second author (NN, LAG, or KC). Using the Template for Intervention Description and Replication [83], 2 authors (LE and NN, LAG, or KC) will independently pilot the standardized data extraction form by extracting 5 studies and then meet to discuss any required modifications to the form to ensure that all relevant data are captured, such as the following:

1. Publication details (study authors, year published) and study details (country, setting, sample size, and design)
2. Participant characteristics (age, gender, and sociodemographic information, including socioeconomic status and geographical remoteness)
3. Intervention characteristics (mode of delivery, duration and frequency of program, underpinning theory, material and components, and targeted risk behavior)
4. Comparison group characteristics
5. Primary and secondary outcomes
6. Measurement tools

The corresponding authors of the published articles will be contacted if additional information that was not reported is required.

### Risk of Bias

The risk of bias of the included studies will initially be judged by one independent reviewer (LE) using the Cochrane Revised Risk of Bias Tool [84]. Sources of bias covered in this tool include the following: randomized allocation to groups, allocation concealment, blinding of participants and personnel, blinding outcome, handling of incomplete data, selective reporting, and other biases not covered. A second reviewer (NN, LAG, or KC) will also rate the risk of bias of the included studies, with any inconsistencies resolved through consultation.

### Analysis

A narrative analysis will be adopted to synthesize the study findings from the included studies. To begin, one reviewer (LE) will tabulate the following results to compare study components and findings: sample characteristics (eg, location, socioeconomic status, gender, and age); risk behavior targeted; intervention content and components (including duration and delivery method); underpinning theory; and primary and secondary outcome effect sizes. The quality of the body of evidence will be independently rated by 2 reviewers (LE and NN, LAG, or KC) using the Grading of Recommendations Assessment, Development and Evaluation framework [85]. LE will then follow the UK Economic and Social Research Council guidance for narrative synthesis in systematic reviews [86], identify themes and factors, and subsequently, summarize the studies in a narrative synthesis.

## Results

As of December 2021, title and abstract screening of 3216 articles was completed, and full-text review was underway. The results will be summarized in a narrative synthesis. The systematic review is expected to be completed and submitted for publication in 2022.

## Discussion

Disadvantaged adolescents, such as those with lower socioeconomic status or living in geographically remote areas, may be more vulnerable to experiencing greater chronic disease burden than their counterparts, as evidenced by the disproportionate levels of chronic disease burden among disadvantaged adult populations [1-6]. Consumption-related chronic disease risk behaviors, such as poor diet, alcohol use, tobacco smoking, and vaping, tend to be greater among these populations than their counterparts [27-32,39,40]. In order to reduce this burden, prevention and early intervention is critical. Several systematic reviews have supported the efficacy of universal eHealth interventions in targeting diet and alcohol and tobacco use among adolescents [75-78]; however, it is unclear whether eHealth interventions adequately serve adolescents living in geographically remote or lower socioeconomic areas. In light of this, this review is the first to systematically examine and synthesize evidence on eHealth interventions targeting disadvantaged adolescents (aged 10-19 years) from socioeconomically disadvantaged backgrounds in preventing poor diet, alcohol use, tobacco smoking, and vaping. We expect that literature on eHealth interventions focused on preventing vaping among disadvantaged adolescents may be limited given that its use has only become more common over the past decade, unlike the other behaviors covered in this review. The results from this systematic review will provide valuable knowledge on the important intervention components of effective eHealth interventions and guide the development of tailored eHealth interventions that are better able to prevent and reduce health risk behaviors among these populations. Ultimately, addressing these health risk behaviors to reduce the vulnerabilities of disadvantaged populations [19] has the potential to provide positive benefits to overall health and narrow the inequalities in health. The results from this review will be disseminated through peer-reviewed journals and conferences to help guide future research projects in this area.

## Appendix

Multimedia Appendix 1 PRISMA-P (Preferred Reporting Items for Systematic Review and Meta-Analysis Protocols) checklist.

Multimedia Appendix 2 Cochrane Database of Systematic Reviews (CDSR) search strategy.

Multimedia Appendix 3 Cochrane Central Register of Controlled Trials (CENTRAL) search strategy.

Multimedia Appendix 4 PROSPERO search strategy.

Multimedia Appendix 5 MEDLINE (Ovid) search strategy.

Multimedia Appendix 6 Embase (Ovid) search strategy.

Multimedia Appendix 7 Scopus search strategy.

Multimedia Appendix 8 Psycinfo (Ovid) search strategy.

## Abbreviations

PRISMA-P: Preferred Reporting Items for Systematic Reviews and Meta-Analysis Protocols
SSB: sugar-sweetened beverage
