# Supplementary material for: eHealth Interventions Targeting Poor Diet, Alcohol Use, Tobacco Smoking, and Vaping Among Disadvantaged Youth: Protocol for a Systematic Review
Source: JMIR Res Protoc. 2022 May 13;11(5):e35408. doi: 10.2196/35408 (PMC9143768; doi:10.2196/35408)
Supplement: Multimedia Appendix 2 [file resprot_v11i5e35408_app2.pdf]

Table S1. Cochrane Database of Systematic Reviews (CDSR) from 1947 to February 2022

Number	Search term
1	ehealth or mhealth or “electronic health” or mobile health or telemedicine or telehealth
2	MeSH descriptor: [Telemedicine] explode all trees
3	#1 OR #2
4	teen* OR adolescen* OR child* OR “young adult”
5	diet*
6	nutrition*
7	alcohol*
8	MeSH descriptor: [Alcoholic beverages] explode all trees
9	smoking*
10	MeSH descriptor: [Smoking] explode all trees
11	cigarette*
12	MeSH descriptor: [Tobacco Products] explode all trees
13	vaping
14	MeSH descriptor: [Vaping] explode all trees
15	#5 OR #6 OR #7 OR #8 OR #9 OR #10 OR #11 OR #12 OR #13 OR #14
16	“socioeconomic status”
17	MeSH descriptor: [Social Class] explode all trees
18	MeSH descriptor: [Socioeconomic Factors] explode all trees
19	“low socioeconomic”
20	poor*
21	MeSH descriptor: [Working Poor] explode all trees
22	“low income”
23	rural
24	MeSH descriptor: [Rural Health] explode all trees
25	MeSH descriptor: [Rural Population] explode all trees
26	regional* OR remote*
27	#16 OR #17 OR #18 OR #19 OR #20 OR #21 OR #22 OR #23 OR #24 OR #25 OR #26
28	#3 AND #15 AND #27
