# Supplementary material for: eHealth Interventions Targeting Poor Diet, Alcohol Use, Tobacco Smoking, and Vaping Among Disadvantaged Youth: Protocol for a Systematic Review
Source: JMIR Res Protoc. 2022 May 13;11(5):e35408. doi: 10.2196/35408 (PMC9143768; doi:10.2196/35408)
Supplement: Multimedia Appendix 4 [file resprot_v11i5e35408_app4.pdf]

**Table S3.** Sample PROSPERO search strategy

| Number | Search term |
|---|---|
| 1 | ehealth AND (alcohol_substance_misuse_abuse OR child_health OR Health inequalities/health equity OR Public health including social determinants of health):HA |
