# Supplementary material for: eHealth Interventions Targeting Poor Diet, Alcohol Use, Tobacco Smoking, and Vaping Among Disadvantaged Youth: Protocol for a Systematic Review
Source: JMIR Res Protoc. 2022 May 13;11(5):e35408. doi: 10.2196/35408 (PMC9143768; doi:10.2196/35408)
Supplement: Multimedia Appendix 5 [file resprot_v11i5e35408_app5.pdf]

Table S4. Sample MEDLINE (Ovid) search strategy from 1946 to February 2022

Number	Search term
1	(ehealth or mhealth or electronic health or mobile health or telemedicine or telehealth).mp.
2	Telemedicine/
3	1 or 2
4	teen*.mp.
5	adolescen*.mp. or Adolescent/
6	child*.mp. or Child/
7	young adult*.mp. or Young Adult/
8	4 or 5 or 6 or 7
9	diet*.mp. or Diet/
10	nutrition.mp.
11	alcohol.mp.
12	Alcoholic Beverages/
13	smoking.mp. or Smoking/
14	cigarette.mp. or Tobacco Products/
15	vaping.mp. or Vaping/
16	9 or 10 or 11 or 12 or 13 or 14 or 15
17	socioeconomic status.mp. or Social Class/
18	Socioeconomic Factors/ or low socioeconomic.mp.
19	poor.mp. or Working Poor/
20	low income.mp.
21	rural.mp. or Rural Health/ or Rural Population/
22	(regional or remote).mp.
23	17 or 18 or 19 or 20 or 21 or 22
24	3 and 8 and 16 and 23
