# Supplementary material for: eHealth Interventions Targeting Poor Diet, Alcohol Use, Tobacco Smoking, and Vaping Among Disadvantaged Youth: Protocol for a Systematic Review
Source: JMIR Res Protoc. 2022 May 13;11(5):e35408. doi: 10.2196/35408 (PMC9143768; doi:10.2196/35408)
Supplement: Multimedia Appendix 7 [file resprot_v11i5e35408_app7.pdf]

Table S6. Scopus search strategy from 1970 to February 2022

Number	Search term
1	ehealth OR mhealth OR "electronic health" OR "mobile health" OR telemedicine OR telehealth
2	AND
3	teen OR adolescent OR child OR "young adult"
4	AND
5	diet OR nutrition OR alcohol OR "alcoholic beverages" OR smoking OR cigarette OR "tobacco products" OR vaping
6	AND
7	"socioeconomic status" OR "social class" OR "socioeconomic factors" OR "low socioeconomic" OR poor OR "working poor" OR "low income" OR rural OR "rural health" OR "rural population" OR regional OR remote
