# Supplementary material for: eHealth Interventions Targeting Poor Diet, Alcohol Use, Tobacco Smoking, and Vaping Among Disadvantaged Youth: Protocol for a Systematic Review
Source: JMIR Res Protoc. 2022 May 13;11(5):e35408. doi: 10.2196/35408 (PMC9143768; doi:10.2196/35408)
Supplement: Multimedia Appendix 8 [file resprot_v11i5e35408_app8.pdf]

**Table S7.** Psycinfo (Ovid) search strategy from 1806 to February 2022

| Number | Search term |
|---|---|
| 1 | (ehealth or mhealth or electronic health or mobile health or telemedicine or telehealth).mp. |
| 2 | Telemedicine/ |
| 3 | 1 or 2 |
| 4 | teen*.mp. |
| 5 | adolescen*.mp. or Adolescent/ |
| 6 | child*.mp. or Child/ |
| 7 | young adult*.mp. or Young Adult/ |
| 8 | 4 or 5 or 6 or 7 |
| 9 | diet*.mp. or Diet/ |
| 10 | nutrition.mp. |
| 11 | alcohol.mp. |
| 12 | Alcoholic Beverages/ |
| 13 | smoking.mp. or Smoking/ |
| 14 | cigarette.mp. or Tobacco Products/ |
| 15 | vaping.mp. or Vaping/ |
| 16 | 9 or 10 or 11 or 12 or 13 or 14 or 15 |
| 17 | socioeconomic status.mp. or Social Class/ |
| 18 | Socioeconomic Factors/ or low socioeconomic.mp. |
| 19 | poor.mp. or Working Poor/ |
| 20 | low income.mp. |
| 21 | rural.mp. or Rural Health/ or Rural Population/ |
| 22 | (regional or remote).mp. |
| 23 | 17 or 18 or 19 or 20 or 21 or 22 |
| 24 | 3 and 8 and 16 and 23 |
